# Diagnostic utility of lipocalin 2 and metalloproteinase 9 levels in early-stage endometrial cancer

**DOI:** 10.1177/18758592241290951

**Published:** 2025-01-17

**Authors:** Songül Ünüvar, Rauf Melekoğlu, Hande Yüce, Nesibe Zeyveli Çelik, Ezgi Bulut Okumuş, Serhat Toprak, Kevser Tanbek, Şeyma Yaşar, Ayşegül Doğan, Neşe Başak Türkmen, Ercan Yılmaz, Süleyman Sandal

**Affiliations:** 1 Department of Pharmaceutical Toxicology, Faculty of Pharmacy, İnönü University, Malatya, Türkiye; 2 Department of Obstetrics and Gynecology, Faculty of Medicine, İnönü University, Malatya, Türkiye; 3 Department of Genetics and Bioengineering, Faculty of Engineering, Yeditepe University, Istanbul, Türkiye; 4 Department of Pathology, Faculty of Medicine, İnönü University, Malatya, Türkiye; 5 Department of Physiology, Faculty of Medicine, İnönü University, Malatya, Türkiye; 6 Department of Biostatistics and Medical Informatics, Faculty of Medicine, İnönü University, Malatya, Türkiye

**Keywords:** Endometrial cancer, lipocalin, biomarker, MMP9, TIMP1, ferritin

## Abstract

**Background:**

Endometrial cancer (EC) is the fourth most common gynecologic malignancy among women. Histopathologic examination is considered gold-standard for diagnosis of EC. However, these examinations sometimes not be useful in distinguishing early stage types of EC.

**Objectıves:**

The current study aimed to investigate the clinicopathological significance of Lipocalin-2 (LCN2), matrix metalloproteinase-9 (MMP9), and ferritin in tumor progression.

**Methods:**

A total of 98 patients (55 women newly diagnosed with early-stage endometrial cancer [study group] and 43 women with benign endometrial pathologies [control group]) were enrolled.

**Results:**

There was a significant difference between diagnosis (p < 0.001), surgical procedure (p < 0.001), pathology (p = 0.002), stage (p < 0.001), lymphovascular invasion (LVI) (p = 0.002), myometrial invasion (p < 0.001), and staining intensity (p < 0.001), MMP9 (p = 0.023), LCN2 (p < 0.001), glucocorticoid (GC) (p = 0.048), tumor necrosis factor-alpha (TNF-α) (p = 0.044), menopause duration (p = 0.001), body weight (p < 0.001), and body mass index (BMI) (p < 0.001) were found to be higher, and ferritin levels (p = 0.047) were lower in the endometrial adenocarcinoma group compared to the benign endometrial pathologies.

**Conclusıon:**

LCN2, MMP9, and ferritin are practical markers in early cases of endometrial cancer. Serum LCN2 and MMP9 levels may be good clinical tools for the auxiliary diagnosis of early-stage endometrial cancer. Ferritin was also significantly sensitive. Therefore, detecting these markers together may be more beneficial for cancer diagnosis.

## Introduction

Endometrial cancer (EC) is the most frequently diagnosed gynecological malignancy, with its incidence rising globally.^1^ Lipocalin-2 (LCN2), known as siderocalin, uterocalin, oncogene 24p3, or transferrin, is a secreted glycoprotein belonging to the adipokine superfamily.^2^ LCN2 protein is secreted by epithelial cells, macrophages, neutrophils, and tumor cells.^3–6^ Members of the lipocalin protein family are characterized by their ability to bind small hydrophobic molecules and specific cell surface receptors to form macromolecular complexes.^7^

LCN2 is linked to several physiological roles, including regulating macroautophagy/autophagy, transporting hydrophobic ligands across the cell membrane, maintaining iron homeostasis during physiological and inflammatory processes, regulating the immune response, and enabling epithelial cell differentiation. LNC2 is detected at high levels in plasma, serum, and urine in aggressive cancer subtypes, including metastatic breast cancer, colorectal cancer, pancreatic, thyroid, ovarian, colon, and bile duct cancers, as well asin various conditions such as acute kidney injury,^3^ pancreatitis,^4^ and preeclampsia.^5^ High levels of LCN2 are associated with increased cell proliferation, cancer cell motility, angiogenesis, cell invasion, and metastasis.^6–8^ Studies have shown that LCN2 secretion is also dependent on sex hormones, especially estrogen, and is associated with the proliferation and apoptosis that occur periodically in the endometrium during the menstrual cycle of premenopausal women.^9^ The most crucial roles of LCN2 in cancer result from its ability to facilitate iron uptake into cancer cells and its ability to form a heterodimer with matrix metalloproteinase-9 (MMP9). Abnormally high LCN2 expression is closely associated with the occurrence and development of endometrial cancer, potentially serving as an essential biomarker for early diagnosis.^10^ Moreover, increased LCN2 expression is associated with aggressive features and poor prognosis in endometrial cancer. An increase in LCN2 levels provides preliminary information related to cancer. Elevated LCN2 production in follicular tissue could serve as a diagnostic biomarker for endometrial cancer. This study aimed to to investigate the relationship between MMP9, LCN2, and ferritin in cancer cell invasion and metastasis. During this evaluation, MMP9 and tissue inhibitor of matrix metalloproteinase-1 (TIMP1) levels were measured. Additionally, the levels of interleukins (IL)-17, IL-1β, IL-8, and tumor necrosis factor-alpha (TNF-α), that stimulate both LCN2 and MMP9, as well as serum ferritin levels, were assessed. In endometrial cancer, the early diagnosis, risk classification, and predictive values of the analyzed parameters were compared with routine markers such as carbohydrate antigen 125 (CA125), carbohydrate antigen 15-3 (CA15-3), carbohydrate antigen 19-9 (CA19-9), and carcinoembryonic antigen (CEA), and the correlations among them were evaluated. Another aim of this study is to assess the gene expression levels of LCN2 and MMP9 in early-stage endometrial cancer and to determine their cellular distribution, as well as their relationship with tumor histological subtype, stage, and grade. LCN2 secretion is dependent on sex hormones, especially estrogens, and is related to periodic proliferation and apoptosis in the endometrium of premenopausal women. Therefore, it was considered useful to also evaluate serum glucocorticoid (GC) and estrogen concentrations.

## Patients and methods

### Study design and setting

The study was conducted at the Department of Gynecology and Obstetrics Unit of İnönü University Faculty of Medicine between November 26, 2021 and September 10, 2022. A total of 98 women met the study criteria. The study group contained 55 subjects with abnormal endometrial pathology, namely endometrial adenocarcinoma. Forty-three subjects who had noncancerous endometrial pathology were placed in the control group, namely those with benign endometrial pathologies. Patients with a recent history of uterine surgery, radiotherapy, and chemotherapy or those taking estrogen and progesterone analogs were excluded. Staging was done according to the International Federation of Gynecology and Obstetrics (FIGO) clinical staging. Stage, myometrial invasion, histological grade, nuclear grade, and staining intensity were recorded. Informed consent was obtained from all participants before the study. The Ethical Committee of Malatya Clinical Research approved the study, İnönü University, dated 10.11.2021, numbered 2021/197. Blood samples were centrifuged at 3500 rpm for 15 min at room temperature, and serum samples were stored at −80 °C until analysis. Tissue samples were taken for immunohistochemical examination during the endometrial biopsy, hysteroscopy, and radical surgery. The histological type of the tumor, differentiation level, and depth of myometrial invasion were determined by immunohistochemistry. The reverse transcription polymerase chain reaction (RT-PCR) method was used in LCN2 and MMP9 gene expression studies.

### ELISA analysis

Ferritin, LCN2, MMP9, TIMP1, IL-17, IL-1β, IL-8, estrogen, GC, and TNF-α were quantified in serum samples using enzyme-linked immunosorbent assay (ELISA) kits (E1702Hu, E1429Hu, E0936Hu, E1236Hu, E0142Hu, E0143Hu, E0089Hu, EA0025Hu, E3643Hu, and E0082Hu; BT-LAB, Birmingham, UK). The measurement ranges of the assays for ferritin, LCN2, MMP9, TIMP1, IL-17, IL-1β, IL-8, estrogen, GC, and TNF-α were 1–400 ng/mL, 20–6000 ng/L, 30–9000 ng/mL, 5–1800 pg/mL, 2–600 ng/L, 20–6000 pg/mL, 5–1000 ng/L, 10–2000 ng/L, 1–300 ng/L, and 3–900 ng/L respectively. The intra-assay coefficients of variation (CV) were less than 10%, and inter-assay CVs were less than 8% for all ELISA kits. Serum CA125, CEA, CA19-9, and CA15-3 were measured with the Roche Cobas E601 system.

### Immunohistochemical evaluation

To detect LCN2 receptors immunohistochemically, 4 μm thick sections were taken from all formalin-fixed and paraffin-embedded tissues. Following deparaffinization and dehydration of the sections, antigen retrieval was performed by microwave boiling in 1 M citrate buffer to reveal the antibody binding sites in the tissue. Immunohistochemical staining steps were performed on the Dako Omnis automatic staining device (Tecan, Canada) according to the protocols established for the LCN2 primary antibody (Rabbit Recombinant Monoclonal Lipocalin-2 / NGAL antibody [EPR 5084] 1:100 Apcam). Evaluations were made on a Nikon Eclipse Ci (Nikon, Nanjing, China) light microscope. In immunohistochemical examination, LCN2 positivity was observed as cytoplasmic staining. A semiquantitative scoring method was used based on staining intensity and the proportion of positively stained cells (cytoplasm or nucleus stained from yellow to dark brown). The section is scored up to 3 points for staining intensity (no positive: 0, weak: 1, moderate: 2, strong: 3) and up to 4 points for the proportion of positive staining cells (≤5% of positive cell proportion: 0, 6–25%: 1, 26–50%: 2, 51–75%: 4). A total score of 0–3 was considered negative, and a total score of 4–12 was considered positive.

### Reverse transcription PCR (RT-PCR) analysis

Total RNA was isolated from patient tissue samples using TRIzol Reagent (Invitrogen, ThermoFisher, USA) according to manufacturer's instructions, and cDNA was synthesized using iScript™ cDNA Synthesis Kit (Bio-Rad, USA). The SYBR Green real-time PCR method was used to determine the gene levels according to a previously described protocol.^11^ MMP9 and LCN2 primers (Table 1) were designed using Primer-BLAST software from the National Center for Biotechnology Information (Bethesda, MD, USA) and synthesized by Sentegen (Ankara, Turkey). β-Actin was used as a housekeeping gene. mRNA expression was normalized to the β-Actin gene and calculated by the 2^−ΔΔCT^ fold method. Results were represented as fold change All Real-time PCR experiments were performed using the CFX96 RT-PCR system (Bio-Rad, USA). Data were statistically analyzed using a t-test. Statistical analysis and graphs of gene analysis were generated using GraphPad Prism version 8.0.1 for Windows (GraphPad Software, San Diego, California USA, www.graphpad.com).

**Table 1.** Primer sequences.

LCN2	F	5’ ACCCTCTACGGGAGAACCAA 3’
	R	5’ CAGGGAGGCCCAGAGATTTG 3’
MMP9	F	5’ TTT GCTGCCCCCAGACAGCG 3’
	R	5’ TCGCATGGCCTTCAGCGTGG 3’
β-Actin	F	5’ GACAGGATGCAGAAGGAGATTACT 3’
	R	5’ TGATCCACATCTGCTGGAAGGT 3’

### Statistical analysis

Data were summarized as mean ± standard deviation, median (min-max), and number (percentage). The Kolmogorov-Smirnov test was used to determine compliance with normal distribution. Statistical analyses included the Mann-Whitney U test, independent sample t-test, Pearson Chi-Square test, Yates Corrected Chi-Square Test, and Fisher Exact Chi-Square test, as appropriate. The relationship between variables was evaluated with the Pearson correlation coefficient. Receiver operating characteristic (ROC) analysis was applied to determine classification performances for diagnosis and the most appropriate cut-off points for relevant variables. The Diagnostic Tests and ROC Analysis Software (DTROC), a web-based software developed by İnönü University Faculty of Medicine, Department of Biostatistics and Medical Informatics, was used for ROC analysis. IBM SPSS Statistics 26.0 was used for other analyses. Logistic regression analysis was performed to estimate odds ratios.^12^ A value of P < 0.05 was considered statistically significant.

## Results

### Demographic and clinical features

The groups’ demographic characteristics and clinical features are summarized in Tables 2 and 3.

**Table 2.** Demographic characteristics and clinical features of the control group.

Characteristics		Benign endometrial pathologies (Control group, *n *= 43)
		Median (Min-Max)
Age (years)		49(39–74)
Parity		3(0–7)
Duration of menopause (years)		0(0–20)
		Mean ± SD
Height (cm)		162.74 ± 7.74
Weight (kg)		76.67 ± 11.26
Body mass index (kg/m^2^)		29.03 ± 4.33
Gravida		4 ± 1.81
		Count (%)
Comorbidity	None	26 (60.47%)
	Hypertension	7 (16.28%)
	Diabetes	10 (23.26%)
Family history	Negative	25 (58.14%)
	Positive	18 (41.86%)
Smoking	Negative	34 (79.07%)
	Positive	9 (20.93%)
Diagnosis	Uterine prolapse	7 (16.28%)
	Treatment-resistant menometrorrhagia	12 (27.91%)
	Myoma	16 (37.21%)
	Pelvic mass	6 (13.95%)
	Endometrial hyperplasia	2 (4.65%)
Surgical procedure	TAH	7 (16.28%)
	TAH + BSO	36 (83.72%)
Pathology	Atrophic endometrium	3 (6.98%)
	Endometrial polyp	11 (25.58%)
	Leiomyoma uteri	14 (32.56%)
	Iatrogenic endometrium	3 (6.98%)
	Chronic nonspecific endometritis	3 (6.98%)
	Endometrium under the influence of estrogen	4 (9.30%)
	Proliferative endometrium	5 (11.63%)
Staining intensity	Negative	10 (23.26%)
	Weak	20 (46.51%)
	Strong	13 (30.23%)
Consequent	Negative	37 (88.10%)
	Positive	5 (11.90%)

Abbreviation: BSO, bilateral salpingo-oophorectomy; TAH, total abdominal hysterectomy.

**Table 3.** Demographic characteristics and clinical features of the study group.

Characteristics		Endometrial adenocarcinoma (study group, *n *= 55)
		Median (Min-Max)
Age (years)		56 (37–82)
Parity		3 (0–9)
Duration of menopause (years)		5 (0–30)
		Mean ± SD
Height (cm)		160.65 ± 7.46
Weight (kg)		87.04 ± 11.82
Body mass index (kg/m^2^)		33.83 ± 4.86
Gravida		3.42 ± 2.45
		Count (%)
Comorbidity	None	30 (54.55%)
	Hypertension	10 (18.18%)
	Diabetes	15 (27.27%)
Family history	Negative	39 (70.91%)
	Positive	16 (29.09%)
Smoking	Negative	52 (94.55%)
	Positive	3 (5.45%)
Surgical procedure	TAH + BSO	47 (85.45%)
	TAH + BSO + PLND	2 (3.64%)
	TAH + BSO + PLND + Omentectomy	6 (10.91%)
Pathology	Endometrial adenocarcinoma	34 (61.82%)
	Well-differentiated endometrioid adenocarcinoma	15 (27.27%)
	Moderately differentiated endometrioid adenocarcinoma	5 (9.09%)
	High-grade endometrial adenocarcinoma	1 (1.82%)
Stage	1a	46 (83.64%)
	1b	9 (16.36%)
Histological grade	1	34 (61.82%)
	2	21 (36.36%)
	3	1 (1.82%)
Nuclear grade	1	21 (38.18%)
	2	32 (58.18%)
	3	2 (3.64%)
LVI	Negative	42 (76.36%)
	Positive	13 (23.64%)
Myometrial invasion	None	5 (9.09%)
	Less than 1/2	40 (72.73%)
	More than 1/2	10 (18.18%)
Staining intensity	Weak	13 (24.07%)
	Strong	41 (75.93%)
Consequent	Negative	11 (20.75%)
	Positive	42 (79.25%)

Abbreviations: BSO, bilateral salpingo-oophorectomy; LVI, lymphovascular invasion; PLND, pelvic lymph node dissection; TAH, total abdominal hysterectomy.

The study group is generally older than the control group, which is consistent with the increased risk of endometrial adenocarcinoma with advancing age. Parity is similar between both groups, suggesting that the number of births may not be significantly different between women with benign and malignant endometrial conditions. The study group has a longer duration of menopause, which may be relevant as prolonged estrogen exposure without progesterone can increase the risk of endometrial cancer. The study group has a higher mean weight, suggesting a possible correlation between higher body weight and endometrial cancer. The study group has a higher body mass index (BMI), indicating obesity as a potential risk factor for endometrial adenocarcinoma. Comorbid conditions like hypertension and diabetes are more common in patients with endometrial adenocarcinoma. Family history might not be a significant differentiator between the groups. Smoking is less common in the study group, which is contrary to the typical risk association with cancer, indicating other factors might play a more prominent role in endometrial adenocarcinoma. More extensive surgical interventions in the study group reflect the complexity and severity of endometrial adenocarcinoma. Early-stage and well-differentiated adenocarcinomas are common in the study group, which may have implications for prognosis and treatment strategies. Strong staining intensity is much higher in the study group, suggesting a higher expression of the LCN2 used for staining in endometrial adenocarcinoma. The data indicate significant differences between the control and study groups in terms of age, weight, BMI, comorbidities, and staining intensity. These findings are consistent with known risk factors for endometrial adenocarcinoma, such as older age, higher BMI, and the presence of comorbid conditions like diabetes and hypertension. The higher prevalence of strong staining intensity in the study group suggests potential diagnostic or prognostic biomarkers for endometrial adenocarcinoma. Further analysis and studies are needed to understand the underlying mechanisms and to validate these findings.

### ELISA results

As shown in Table 4, the study group had significantly higher numerical values of demographic characteristics such as average age (p < 0.001), menopause period (p = 0.001), weight (p < 0.001), and BMI (p < 0.001). The study group also had significantly higher levels of MMP9 (p = 0.023), GC (p = 0.048), LCN2 (p < 0.001), staining intensity (p < 0.001), and TNF-α (p = 0.044) compared to the control group. In contrast, ferritin levels (p = 0.047) were lower in the study group. Significant differences were observed between the two groups regarding diagnosis (p < 0.001), surgical procedure (p < 0.001), pathology (p = 0.002), stage (p < 0.001), lymphovascular invasion (LVI) (p = 0.002), myometrial invasion (p < 0.001), and staining intensity (p < 0.001).

**Table 4.** Comparison between the biomarkers and clinicopathological parameters of benign endometrial pathologies and endometrial adenocarcinoma (n = 98).

		Benign endometrial pathologies (Control group, n = 43)		Endometrial adenocarcinoma (Study group, n = 55)	*P* value
Age (years)		49(39–74)		56(37–82)	<0.001
Parity		3(0–7)		3(0–9)	0.073
Duration of menopause (years)		0(0–20)		5(0–30)	0.001
Ca125		14.15(4–244)		14.6(2.9–167.7)	0.670
Ca15-3		14.45(3.7–31)		13.7(3.1–46.9)	0.618
AFP		1.72(0.2–6.4)		2.1(0.6–6.67)	0.393
MMP9 (ng/L)		1317.52(553.21–3973.66)		1495.41(911.59–3336.47)	0.023
IL-17 (ng/L)		119.69(55.89–324.54)		109.76(81.35–264.06)	0.342
GC (ng/L)		35.53(18.46–104.91)		42.66(6.08–117.81)	0.048
LCN2 (ng/L)		987.23(534.52–3193.92)		1290.76(110.86–2405)	<0.001
IL-1β (pg/mL)		917.77(299.95–1754.29)		926.84(327.16–1947.8)	0.653
Ferritin (ng/mL)		62(29.75–172.52)		50.82(30.89–108.5)	0.047
Estrogen (ng/L)		1.73(1.09–2.23)		1.73(1.24–2.24)	0.597
Staining intensity (1-2-3-4)		7.5(1–95)		60(2–100)	<0.001
		Mean ± SD		Mean ± SD	
Gravida		4 ± 1.81		3.42 ± 2.45	0.057
Height (cm)		162.74 ± 7.74		160.65 ± 7.46	0.173
Weight (kg)		76.67 ± 11.26		87.04 ± 11.82	<0.001
BMI (kg/m^2^)		29.03 ± 4.33		33.83 ± 4.86	<0.001
CEA		1.46 ± 0.62		1.69 ± 0.66	0.526
Ca19-9		13.42 ± 10.58		58.96 ± 205.28	0.184
IL-8 (ng/L)		300.75 ± 74.95		335.87 ± 118.55	0.265
TIMP1 (pg/mL)		532.88 ± 167.05		509.31 ± 137.65	0.415
TNF-α (ng/L)		158.89 ± 47.83		190.43 ± 63.28	0.044
		Count (%)		Count (%)	
Comorbidity	None		26 (60.47%)	30 (54.55%)	0.839
	Hypertension		7 (16.28%)	10 (18.18%)	
	Diabetes		10 (23.26%)	15 (27.27%)	
Family history	Negative		25 (58.14%)	39 (70.91%)	0.27
	Positive		18 (41.86%)	16 (29.09%)	
Smoking	Negative		34 (79.07%)	52 (94.55%)	0.045
	Positive		9 (20.93%)	3 (5.45%)	
Diagnosis	Uterine prolapse		7 (16.28%)	0 (0.00%)	<0.001
	Treatment-resistant menometrorrhagia		12 (27.91%)	0 (0.00%)	
	Myoma		16 (37.21%)	0 (0.00%)	
	Pelvic mass		6 (13.95%)	0 (0.00%)	
	Endometrial hyperplasia		2 (4.65%)	0 (0.00%)	
	Endometrial adenocarcinoma		0 (0.00%)	55 (100.00%)	
Surgical procedure	TAH		7 (16.28%)	0 (0.00%)	<0.001
	TAH + BSO		36 (83.72%)	47 (85.45%)	
	TAH + BSO + PLND		0 (0.00%)	2 (3.64%)	
	TAH + BSO + PLND + Omentectomy		0 (0.00%)	6 (10.91%)	
Pathology	Atrophic endometrium		3 (6.98%)	0 (0.00%)	0.002
	Endometrial polyp		11 (25.58%)	0 (0.00%)	
	Leiomyoma uteri		14 (32.56%)	0 (0.00%)	
	Iatrogenic endometrium		3 (6.98%)	0 (0.00%)	
	Chronic nonspecific endometritis		3 (6.98%)	0 (0.00%)	
	Endometrium under the influence of estrogen		4 (9.30%)	0 (0.00%)	
	Proliferative endometrium		4 (9.30%)	0 (0.00%)	
	Endometrium CA		1 (2.33%)	0 (0.00%)	
	Endometrial adenocarcinoma		0 (0.00%)	34 (61.82%)	
	Well-differentiated endometrioid adenocarcinoma		0 (0.00%)	15 (27.27%)	
	Moderately differentiated endometrioid adenocarcinoma		0 (0.00%)	5 (9.09%)	
	High-grade endometrial adenocarcinoma		0 (0.00%)	1 (1.82%)	
Stage	1a		0 (0.00%)	46 (83.64%)	<0.001
	1b		0 (0.00%)	9 (16.36%)	
	2		0 (0.00%)	0 (0.00%)	
	None		43 (100.00%)	0 (0.00%)	
LVI	Negative		43 (100.00%)	42 (76.36%)	0.002
	Positive		0 (0.00%)	13 (23.64%)	
Myometrial invasion	None		43 (100.00%)	5 (9.09%)	<0.001
	Less than 1/2		0 (0.00%)	40 (72.73%)	
	More than 1/2		0 (0.00%)	10 (18.18%)	
Staining intensity	Negative		10 (23.26%)	0 (0.00%)	<0.001
	Weak		20 (46.51%)	13 (24.07%)	
	Strong		13 (30.23%)	41 (75.93%)	
Consequent	Negative		37 (88.10%)	11 (20.75%)	<0.001
	Positive		5 (11.90%)	42 (79.25%)	

Abbreviations: AFP, alpha-fetoprotein; BMI, body mass index; BSO, bilateral salpingo-oophorectomy; Ca125, carbohydrate antigen 125; Ca15-3, carbohydrate antigen 15-3; Ca19-9, carbohydrate antigen 19-9; CEA, carcinoembryonic antigen; GC, glucocorticoid; IL-17, interleukin 17; IL-1β, interleukin 1β; IL-8, interleukin 8; LCN2, lipocalin-2; LVI, lymphovascular invasion; MMP9, matrix metalloproteinase-9; PLND, pelvic lymph node dissection; TAH, total abdominal hysterectomy; TIMP1, tissue inhibitor of metalloproteinase 1; TNF-α, tumor necrosis factor alpha.

The study group is significantly older, indicating that advanced age is a risk factor for endometrial adenocarcinoma. Longer duration of menopause is associated with endometrial adenocarcinoma, likely due to prolonged estrogen exposure without progesterone. Higher weight and BMI in the study group highlight obesity as a significant risk factor for endometrial adenocarcinoma. Elevated MMP9 levels in the study group suggest its role in tumor invasion and metastasis. Higher GC levels may indicate stress or inflammatory responses associated with cancer. Significantly higher LCN2 levels suggest its potential as a biomarker for endometrial adenocarcinoma. Elevated TNF-α levels indicate an inflammatory environment, which is common in cancer progression. Lower ferritin levels could be due to cancer-related iron metabolism alterations. Presence of LVI indicates a more aggressive disease with potential for metastasis. Significant myometrial invasion correlates with disease severity. The data highlight significant differences in demographic characteristics, biomarkers, and clinicopathological parameters between benign endometrial pathologies and endometrial adenocarcinoma. Older age, longer menopause duration, higher weight and BMI, and elevated levels of biomarkers like MMP9, GC, LCN2, and TNF-α are associated with endometrial adenocarcinoma. Pathological features such as lymphovascular invasion, myometrial invasion, and strong staining intensity further differentiate malignant cases from benign ones. These findings underscore the importance of these parameters in diagnosing and understanding the progression of endometrial adenocarcinoma.

**Figure 1.**
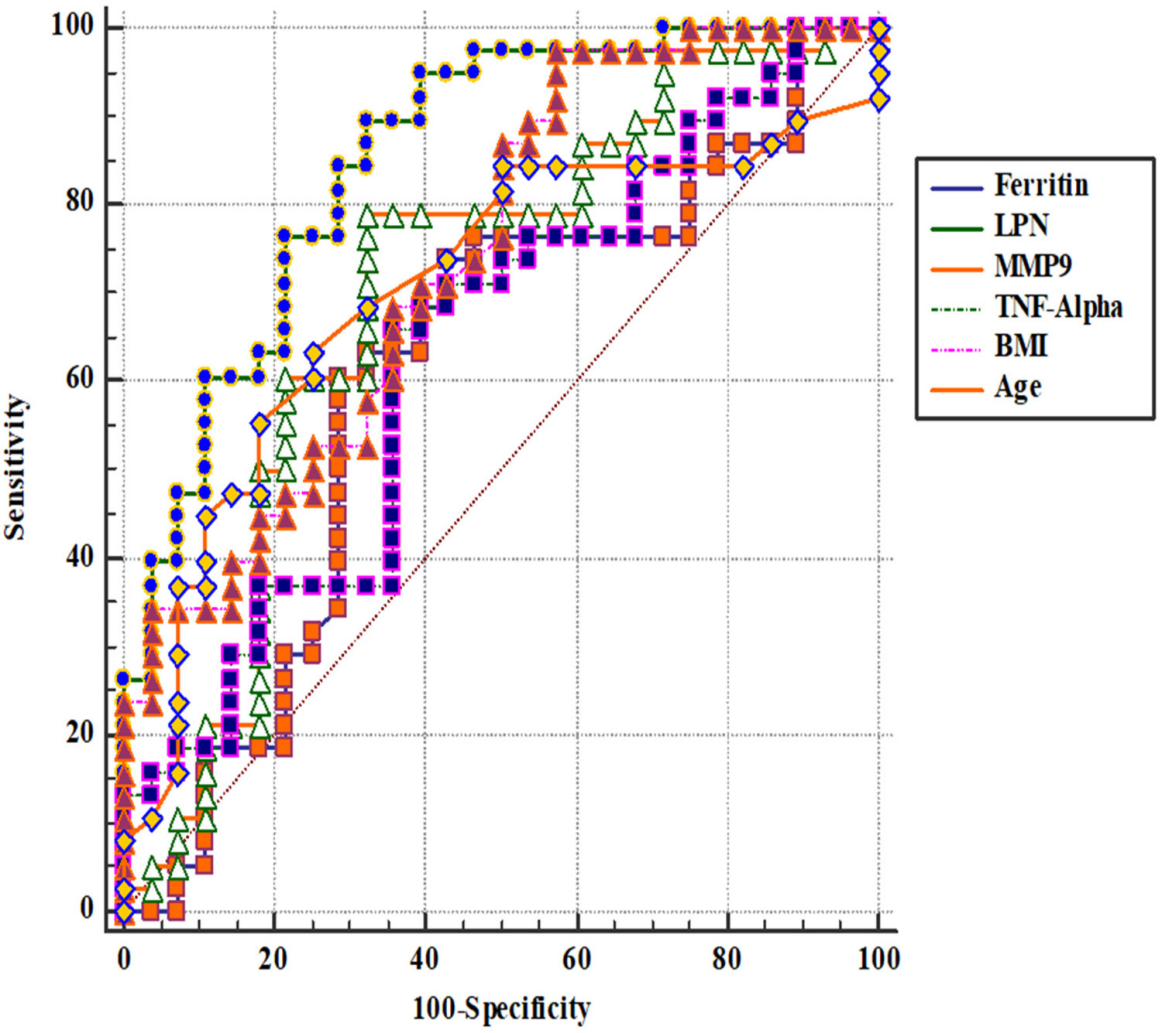
Receiver operating characteristic analysis showing the utility of ferritin, LCN2, MMP9, TNF-α, BMI, and age in patients with endometrial adenocarcinoma.

ROC curve analysis was conducted to assess the ability of biomarkers to predict endometrial cancer (Table 5, Figure 1).

**Table 5.** ROC analysis showing the predictive value of biomarkers for endometrial cancer.

Variables	Cut-off	Sensitivity	Specificity	LR+	LR-	PPV	NPV	AUC (95%CI)	*P* value
Age	54	61.8 (47.7–74.6)	74.4 (58.8–86.5)	2.42	0.51	75.6	60.4	0.705 (0.604–0.793)	<0.001
BMI	27.4	94.6 (84.9–98.9)	41.9 (27.0–57.9)	1.63	0.13	66.7	85.0	0.754 (0.657–0.836)	<0.001
Ca125	10.8	42.6 (28.3–57.8)	75.0 (58.8–87.3)	1.70	0.77	66.7	52.6	0.527 (0.417–0.635)	0.673
Ca15-3	17	40.0 (16.3–67.7)	90.0 (55.5–99.7)	4.00	0.67	85.7	50.0	0.560 (0.349–0.756)	0.623
Ca19-9	9.7	70.7 (54.5–83.9)	50.0 (32.9–67.1)	1.41	0.59	61.7	60.0	0.588 (0.470–0.699)	0.180
CEA	1.7	54.55 (23.4–83.3)	70.0 (34.8–93.3)	1.82	0.65	66.7	58.3	0.582 (0.350–0.790)	0.525
MMP9	1341.5	76.9 (60.7–88.9)	62.5 (43.7–78.9)	2.05	0.37	71.4	69.0	0.658 (0.536–0.766)	0.022
IL-17	108.9	50.0 (33.8–66.2)	70.97 (52.0–85.8)	1.72	0.70	69.0	52.4	0.566 (0.443–0.683)	0.343
GC	36.8	78.6 (63.2–89.7)	59.4 (40.6–76.3)	1.93	0.36	71.7	67.9	0.635 (0.515–0.744)	0.054
LCN2	1035.2	85.0 (70.2–94.3)	59.4 (40.6–76.3)	2.09	0.25	72.3	76.0	0.759 (0.644–0.852)	<0.001
IL-1β	728.3	34.2 (20.1–50.6)	81.3 (63.6–92.8)	1.82	0.81	70.0	49.1	0.531 (0.410–0.649)	0.652
IL-8	475.2	18.9 (8.0–35.2)	100.0 (88.8–100)	-	0.81	100	50.8	0.579 (0.453–0.698)	0.262
Ferritin	54.1	60.5 (43.4–76.0)	75.0 (56.6–88.5)	2.42	0.53	74.2	61.5	0.639 (0.515–0.750)	0.044
TIMP1	460.1	51.4 (34.4–68.1)	67.9 (47.6–84.1)	1.60	0.72	67.9	51.4	0.559 (0.431–0.682)	0.420
TNF-α	162.6	67.5 (50.9–81.4)	64.3 (44.1–81.4)	1.89	0.51	73.0	58.1	0.645 (0.519–0.757)	0.036
Estrogen	1.6	31.7 (18.1–48.1)	78.1 (60.0–90.7)	1.45	0.87	65.0	47.2	0.536 (0.416–0.654)	0.599

Abbreviations: AUC, area under the curve; BMI, body mass index; Ca125, carbohydrate antigen 125; Ca15-3, carbohydrate antigen 15-3; Ca19-9, carbohydrate antigen 19-9; CEA, carcinoembryonic antigen; GC, glucocorticoid; IL-17, interleukin 17; IL-1β, interleukin 1β; IL-8, interleukin 8; LCN2, lipocalin-2; LR−, negative likelihood ratio; LR+, positive likelihood ratio; MMP9, matrix metalloproteinase-9; NPV, negative predictive value; PPV, positive predictive value; TIMP1, tissue inhibitor of metalloproteinase-1; TNF-α, tumor necrosis factor-alpha.

The data indicate that certain biomarkers like BMI, LCN2, and age have significant predictive value for endometrial cancer, while others like Ca125 and IL-8 are less effective. BMI showed the highest sensitivity among all biomarkers, reflecting its strong association with endometrial cancer risk. LCN2 and MMP9 also demonstrated good predictive capabilities, which aligns with their known roles in inflammation and cancer pathology. The age cut-off highlights the increased risk of endometrial cancer in older women. These findings can help in developing diagnostic panels for early detection of endometrial cancer. Combining multiple biomarkers with demographic factors could enhance predictive accuracy and lead to better clinical outcomes. Further studies might focus on validating these cut-offs in larger cohorts and exploring the underlying mechanisms that link these biomarkers with endometrial cancer.

The correlations between the control and study groups are presented in Table 6.

**Table 6.** Correlations between control and study groups.

			Menopose duration	BMI	Ca15-3	MMP9 (ng/L)		IL-17 (ng/L)	GC (ng/L)	LCN2 (ng/L)	IL-1β (pg/mL)	IL-8 (ng/L)	Ferritin (ng/mL)	TIMP1 (pg/mL)	TNF-α (ng/L)	Estrogen (ng/L)
Benign endometrial pathologies	Age	r	0.807	0.379	−0.153		0.255	0.108	0.028	0.322	0.212	0.165	0.136	0.026	0.174	−0.134
		p-value	<0.001	0.012	0.673		0.159	0.562	0.880	0.072	0.244	0.374	0.458	0.896	0.377	0.463
	Menopose duration	r		0.301	−0.243		0.393	0.124	0.227	0.507	0.309	0.206	0.188	−0.016	0.258	0.044
		p-value		0.050	0.499		0.026	0.508	0.212	0.003	0.085	0.266	0.302	0.934	0.184	0.810
	Ca125	r			0.831		−0.147	−0.227	−0.060	−0.107	−0.098	−0.185	−0.103	−0.216	−0.052	0.214
		p-value			0.003		0.447	0.245	0.758	0.581	0.613	0.345	0.593	0.300	0.805	0.265
	CEA	r					−0.471	−0.625	0.327	−0.737	0.285	−0.125	0.180	−0.398	0.745	−0.756
		p-value					0.286	0.134	0.475	0.059	0.535	0.790	0.699	0.377	0.055	0.049
	Ca19-9	r					−0.261	−0.150	−0.238	−0.238	−0.064	−0.141	−0.265	−0.163	−0.056	−0.419
		p-value					0.189	0.464	0.232	0.232	0.750	0.491	0.181	0.458	0.799	0.030
	MMP9 (ng/L)	r						0.891	0.584	0.829	0.660	0.721	0.562	0.871	0.660	0.275
		p-value						<0.001	<0.001	<0.001	<0.001	<0.001	0.001	<0.001	<0.001	0.127
	IL-17 (ng/L)	r							0.465	0.630	0.596	0.702	0.313	0.741	0.725	0.134
		p-value							0.008	<0.001	<0.001	<0.001	0.086	<0.001	<0.001	0.472
	GC (ng/L)	r								0.624	0.585	0.608	0.372	0.418	0.815	0.298
		p-value								<0.001	<0.001	<0.001	0.036	0.027	0.000	0.097
	LCN2 (ng/L)	r									0.737	0.834	0.458	0.614	0.640	0.171
		p-value									<0.001	<0.001	0.008	0.001	<0.001	0.350
	IL-1β (pg/mL)	r										0.803	0.382	0.420	0.754	−0.045
		p-value										<0.001	0.031	0.026	<0.001	0.809
	IL-8 (ng/L)	r											0.288	0.534	0.856	0.047
		p-value											0.116	0.003	<0.001	0.803
	Ferritin (ng/mL)	r												0.455	0.371	0.327
		p-value												0.015	0.052	0.068
	TIMP1 (pg/mL)	r													0.425	0.109
		p-value													0.030	0.582
Endometrial adenocarcinoma	Age	r	0.831	0.249	−0.299		−0.046	−0.090	−0.068	−0.018	−0.032	0.185	0.019	0.046	0.002	−0.056
		p-value	<0.001	0.066	0.279		0.779	0.579	0.669	0.913	0.840	0.272	0.912	0.787	0.991	0.727
	Ca125	r			0.627		0.009	−0.092	0.018	−0.037	−0.075	−0.146	−0.077	−0.170	0.058	−0.094
		p-value			0.012		0.960	0.598	0.914	0.832	0.664	0.425	0.669	0.351	0.739	0.587
	CEA	r					−0.469	−0.763	−0.311	−0.661	−0.782	−0.393	−0.124	−0.585	−0.550	0.203
		p-value					0.242	0.017	0.416	0.053	0.022	0.336	0.770	0.128	0.158	0.600
	Ca19-9	r					−0.103	0.065	−0.124	−0.087	0.021	0.514	−0.020	0.011	−0.063	0.016
		p-value					0.594	0.732	0.499	0.646	0.912	0.006	0.921	0.956	0.742	0.934
	MMP9 (ng/L)	r						0.680	0.772	0.755	0.573	0.163	0.382	0.674	0.677	0.012
		p-value						<0.001	<0.001	<0.001	<0.001	0.341	0.018	<0.001	<0.001	0.943
	IL-17 (ng/L)	r							0.540	0.741	0.630	0.346	0.462	0.697	0.751	0.014
		p-value							<0.001	<0.001	<0.001	0.036	0.004	<0.001	<0.001	0.930
	GC (ng/L)	r								0.731	0.612	0.291	0.564	0.559	0.715	0.001
		p-value								<0.001	<0.001	0.081	<0.001	<0.001	<0.001	0.995
	LCN2 (ng/L)	r									0.619	0.267	0.537	0.686	0.651	0.094
		p-value									<0.001	0.110	0.001	<0.001	<0.001	0.564
	IL-1β (pg/mL)	r										0.351	0.348	0.514	0.684	0.152
		p-value										0.036	0.032	0.001	<0.001	0.349
	Ferritin (ng/mL)	r												0.340	0.412	0.271
		p-value												0.040	0.010	0.099
	TIMP1 (pg/mL)	r													0.559	0.033
		p-value													<0.001	0.844

Abbreviations: BMI, body mass index; Ca125, carbohydrate antigen 125; Ca15-3, carbohydrate antigen 15-3; Ca19-9, carbohydrate antigen 19-9; CEA, carcinoembryonic antigen; GC, glucocorticoid; IL-17, interleukin 17; IL-1β, interleukin 1β; IL-8, interleukin 8; LCN2, lipocalin-2; MMP9, matrix metalloproteinase-9; TIMP1, tissue inhibitor of metalloproteinase-1; TNF-α, tumor necrosis factor-alpha.

Both groups show strong correlations between inflammatory markers, indicating a shared inflammatory pathway. The control group shows more significant correlations with menopause duration, whereas the cancer group shows significant correlations with age and specific cancer markers like Ca125. LCN2 and MMP9 consistently show strong correlations across both groups, suggesting their pivotal role in endometrial pathologies. Ferritin shows significant correlations in the cancer group, indicating its potential role as a cancer biomarker. These correlations provide insight into the biological mechanisms underlying benign and malignant endometrial conditions and could guide future research and clinical diagnostics.

The results of the logistic regression analysis, which included variables with a p-value below 0.25 in the differences between the two groups (TNF-α, IL-8, Ca19-9, ferritin, GC, LCN2, and MMP9), are given in Table 7. When the ferritin value (p = 0.023) increases by 1 unit, the risk of endometrial adenocarcinoma decreases by 0.097 times, meaning that a 1-unit increase in ferritin value decreases the risk of endometrial adenocarcinoma by 9.7%. Conversely, when the LCN2 value (p = 0.009) increases by 1 unit, the risk of endometrial adenocarcinoma increases by 1.010 times.

**Table 7.** Odds ratios for endometrial cancer risk calculated by multivariate regression analysis.

95% confidence interval for the odds ratio				
Variables	Odds ratio	Lower	Upper	*P* value
TNF-α (ng/L)	1.001	0.967	1.036	0.966
IL-8 (ng/L)	1.005	0.994	1.017	0.366
Ca19-9	1.004	0.981	1.026	0.762
Ferritin (ng/mL)	0.903	0.827	0.986	0.023
GC (ng/L)	1.085	0.937	1.257	0.274
LCN2 (ng/L)	1.010	1.002	1.017	0.009
MMP9 (ng/L)	0.996	0.990	1.002	0.164
Constant	0.006	0.967	1.036	0.058

Abbreviations: Ca19-9, carbohydrate antigen 19-9; GC, glucocorticoid; IL-8, interleukin 8; LCN2, lipocalin-2; MMP9, matrix metalloproteinase-9; TNF-α, tumor necrosis factor-alpha.

Ferritin is a variable with a statistically significant protective effect against endometrial adenocarcinoma. This finding aligns with previous research suggesting ferritin's role in cancer biology. LCN2 shows a significant association with increased risk, indicating its potential role as a biomarker for endometrial cancer. Other variables, including TNF-α, IL-8, Ca19-9, GC, and MMP9, do not show statistically significant associations, suggesting their limited predictive value for endometrial adenocarcinoma in this multivariate context. These results highlight the importance of ferritin and LCN2 in the context of endometrial adenocarcinoma and suggest potential pathways for further research and therapeutic targeting.

### İmmunohistochemical results

**Figure 2.**
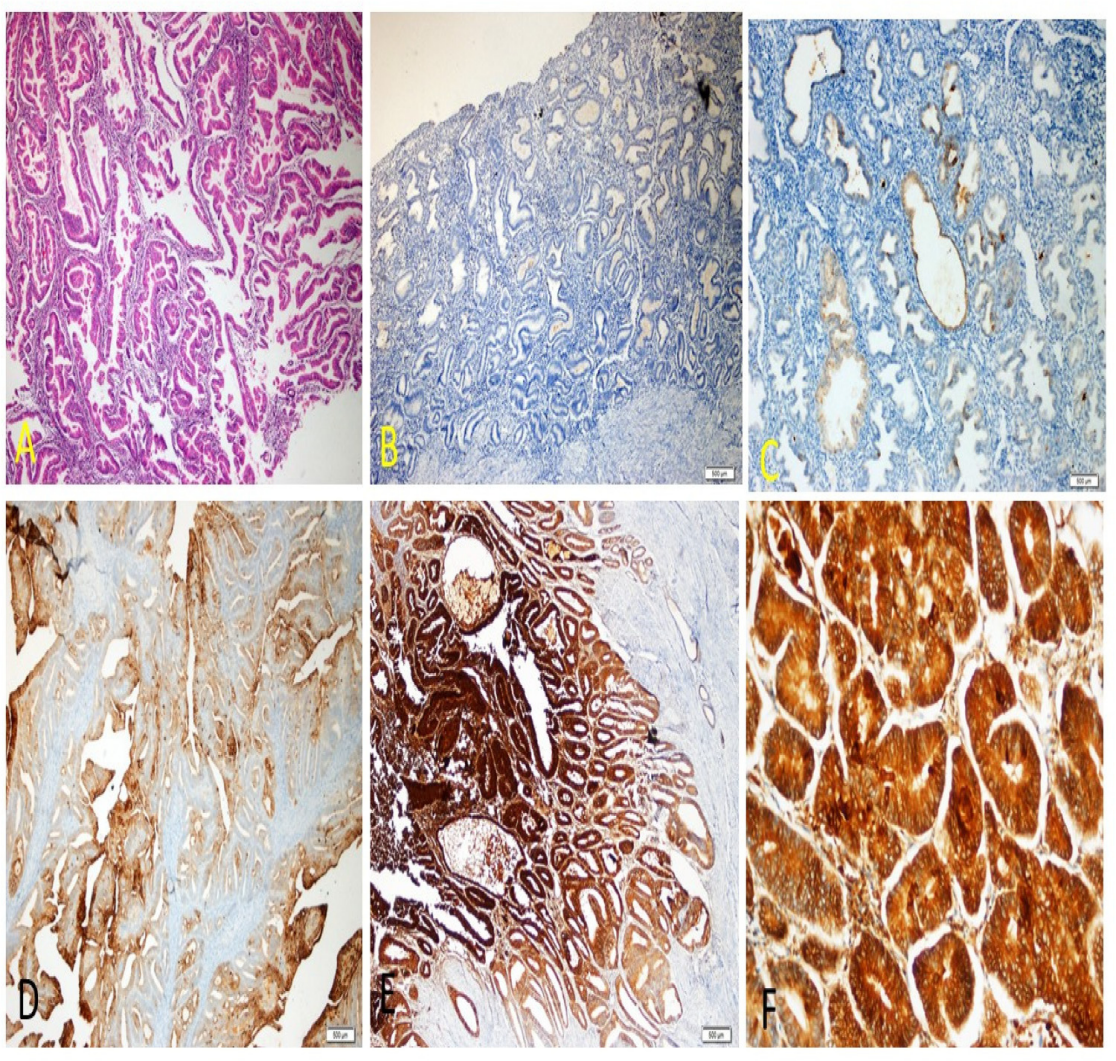
(a) Endometrioid carcinoma showing irregular gland proliferation lined with atypical epithelium laid back to back (H&amp; E x40) (b) LCN2 negative secretory glands (LCN2 x40) (c) Weak staining (LCN2 x100) (d) Medium intensity staining (LCN2 x100) (e) Strong staining (LCN2 x40) (f) Strong staining (LCN2 x200).

The positive expression of LCN2 in endometrial tissues (Figure 2).

Weak LCN2 expression is observed in some areas, suggesting that LCN2 is present in low amounts. The weak staining may be localized to certain epithelial cells within the endometrial glands, indicating early or less aggressive stages of carcinoma. Medium intensity staining indicates a moderate level of LCN2 expression. This staining is likely present in the epithelial cells lining the endometrial glands. The moderate expression could be associated with progression towards malignancy, with LCN2 localized within the cytoplasm of the epithelial cells. Strong LCN2 expression is seen at lower magnification, suggesting a higher level of LCN2 in the more aggressive or advanced stages of carcinoma. The strong staining likely indicates substantial cytoplasmic and possibly membranous localization of LCN2 in the epithelial cells of the endometrial glands. In summary, the specific cellular compartments exhibiting LCN2 expression based on the immunohistochemical results are primarily the cytoplasm of the epithelial cells lining the endometrial glands. The intensity of LCN2 expression varies with the stage and aggressiveness of the carcinoma, with stronger expression indicating more advanced malignancy.

### RT-PCR results

**Figure 3.**
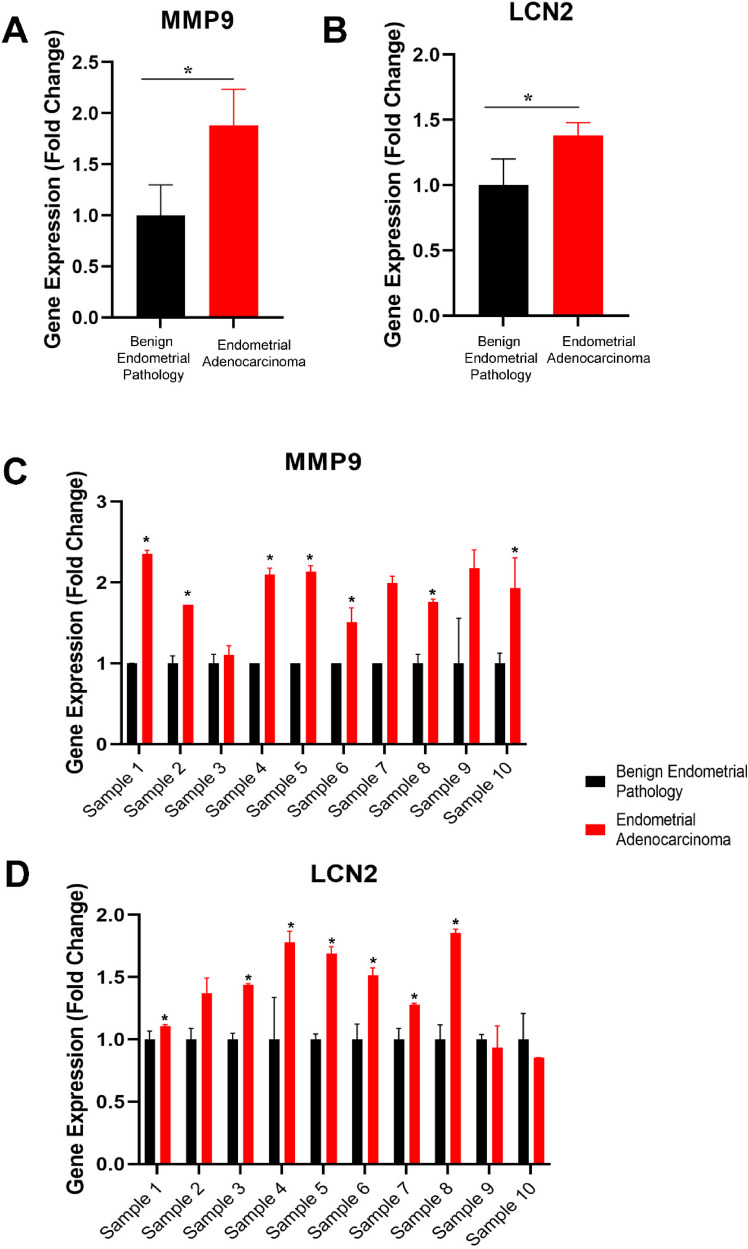
Gene expression analysis of MMP9 and LCN2 for 10 selected patient samples (endometrial adenocarcinoma and benign endometrial pathology) is shown. The average fold change mRNA expression for the 10 selected patient samples is presented for (a) MMP9 and (b) LCN2. Individual gene expression analyses (fold change) of 10 selected patient samples are shown for (c) MMP9 and (d) LCN2. * P < 0.05.

Avarage gene expression of MMP9 and LCN2 showed a significant increase in endometrial adenocarcinoma samples compared to benign endometrial pathology samples (Figure 3(a) and (b)). The average gene expression data for MMP9 in 10 selected patient samples (Figure 3(a)) demonstrated approximately a 1.9-fold increase in the endometrial adenocarcinoma group. LCN2 showed an approximately 1.4-fold increase in the endometrial adenocarcinoma group (Figure 3(b)). 8 of the 10 selected patient samples showed a significant increase in MMP9 gene expression (Figure 3(c)), and 7 of the 10 selected patient samples showed a substantial increase in LCN2 gene expression (Figure 3(d)).

## Discussion

In vitro studies have shown that the expression of LCN2 is associated with the degree of tumor differentiation in endometrial cancer cell lines (Ishikawa, HEC-1-A, and KLE). It is suggested that LCN2 could be a helpful marker for the early detection of endometrial cancer lesions.^1^ LCN2 is mainly expressed in tumor cells originating from the glandular epithelium. Yang et al. reported that LCN2 could promote EMT in human breast cancer cells.^13^ Hanai et al. demonstrated that LCN2 inhibits the invasion, proliferation, and metastasis of murine breast cancer 4T1-Ras-transformed cells by promoting cell EMT.^14^ Although the effects of LCN2 on the endometrium are not fully understood, Lin and colleagues observed in their study on endometrial cancer cell lines that after 24 h of incubation, there was a decrease in apoptosis, changes in cell proliferation, and a reduction in cytokine secretion, particularly the activation of IL-8 and IL-6. A relationship was found between high levels of LCN2 and serum glucocorticoid and estrogen concentrations in patients.^9^ In this latest study, high serum LCN2 levels and an increase in gene expression were found in patients with endometrial cancer. According to ROC analysis data, it was determined that LCN2 could be a significant biomarker and that a 1-unit increase in LCN2 value increases the risk of endometrial adenocarcinoma by 1.010 times. Among all the markers analyzed with LCN2, a positive correlation was found, except for estrogen and IL-8 in healthy and diseased women. Given its potential as an early detection marker, further research could focus on developing LCN2-based diagnostic tests for endometrial cancer. This could involve creating assays to measure serum LCN2 levels in at-risk populations. Understanding the mechanisms by which LCN2 influences tumor behavior (e.g. promoting EMT, reducing apoptosis) could lead to targeted therapies that inhibit LCN2 function, thereby potentially slowing tumor progression and improving patient outcomes. Since LCN2 is implicated in multiple cancer types, comparative studies across different cancers could elucidate common pathways and lead to broader applications of LCN2-based diagnostics and treatments. The correlation with glucocorticoid and estrogen levels suggests a hormonal regulation of LCN2. Investigating these interactions further could reveal novel insights into the hormonal influences on cancer progression and the potential for combined hormonal and LCN2-targeted therapies. Combining LCN2 with other biomarkers (like IL-8, IL-6) could enhance diagnostic accuracy and provide a more comprehensive understanding of the tumor microenvironment and immune interactions in endometrial cancer.

Recent studies on endometrial cancer have shown that LCN2 plays a role in tumor progression. A study indicatedthat LCN2 is the gene with the greatest expression variation between carcinomas and benign tissues, such as hyperplasia and normal endometrium.^15^ In endometrial cancer cell lines, IL-8 was found to be the cytokine most induced by LCN2. Through this mechanism, LCN2 enhances cell survival by preventing apoptosis and increasing cell migration.^9^ The cut-off point for the serum concentration of LCN2 to distinguish benign endometrial changes from endometrial cancer was determined to be 160 ng/mL. Additionally, thesensitivity of the LCN2 protein was found to be higher than that of the HE4 and Ca125 markers across the entire patient population.^16^ While the specificity of LCN2 is not strong as that of HE4, the specificity of LCN2 was found to be higher than that of HE4 (87% and 85%, respectively) in the postmenopausal patient group.^17^ Another study investigating the relationship between LCN2 expression and angiogenesis markers found that LCN2 was not associated with the expression of VEGF-C, VEGF-D, or bFGF2, but showed a significant relationship with VEGF-A expression. Additionally, LCN2 was suggested to be associated with several markers related to EMT (E-cadherin, N-cadherin, P-cadherin, β-catenin) or vascular invasion (tumor).^7^ In our study, we found that tumor markers such as Ca125, CEA, and Ca19-9 were associated with interleukins and estrogen and showed a significant correlation.

Iron promotes cancer initiation, growth, and metastasis. Therefore, a positive relationship has been reported between high dietary iron intake, increased body iron stores, hereditary hemochromatosis, and increased cancer risk. Elevated iron levels lead to tumor growth.^18^ Consequently, reducing iron levels or preventing iron intake using iron chelators can suppress tumor growth. Although the pro-apoptotic property of iron-free LCN2 is not fully understood, it is thought to trigger apoptosis by downregulating Bcl2 and upregulating Bax. It induces iron deficiency by promoting iron efflux from cancer cells, leading to cell death as a result.^19^ Numerous cancer studies have found that LCN2 increases cancer cell survival, growth, and metastasis and enhances cellular resistance to iron-induced toxicity and chemotherapeutics, thus facilitating tumor formation. Significantly reduced ferrous iron (Fe^2+^) content was detected in LCN2-deficient tumors. This highlights the role of LCN2 in facilitating iron uptake in tumors.^20^ The pathogenesis of endometrial cancer is complex and not fully understood. In our study, we found a decrease in ferritin levels. Based on the results of the ROC analysis, we can suggest that ferritin could be a significant marker for endometrial cancer. For each unit increase in ferritin, the risk of endometrial adenocarcinoma decreases by 9.7%. Among all the markers analyzed, only ferritin decreased in the cancer group. Interestingly, it showed a positive correlation with MMP9, LCN2, TIMP1, TNF-α, and interleukins.^21^ Less than one-third of patients diagnosed with gynecologic cancer initially present with anemia. However, within six months of diagnosis, the prevalence increases, with more than half of the patients experiencing anemia. The presence and severity of anemia Are significantly associated with the cancer stage, with the prevalence and degree of anemia increasing in advanced-stage diseases.

No studies have been found that evaluate the role of the TME cytokines, such as IL-17, IL1β, IL-8, and TNF-α, in stimulating LCN2 and MMP9 in endometrial cancer. Additionally, no research has been encountered that assesses the relationship between LCN2 and ferritin in endometrial cancer. Previous studies have evaluated serum ferritin levels exclusively in ovarian cancer among gynecological cancers.Most studies on the relationship between LCN2 and endometrial cancer have focused on MMP9-mediated cancer cell invasion and metastasis. However, despite being mentioned in reviews, the mechanisms through which iron facilitates tumor progression have not been investigated. In this respect, our study is a mechanistic investigation into previously unexplored mechanisms. If these mechanisms are elucidated, new strategies, for cancer treatment, such as the use of MMP9 inhibitors, could be proposed.In our previous study, we found that serum levels of CEA, periostin, and IDO were significantly higher lin women with endometrial cancer compared to healthy women.^22^ Understanding the molecular factors associated with prognosis, especially in patients with advanced and recurrent endometrial cancer, is essential for developing effective treatment strategies. Therefore, in recent years, our research team has focused on identifying biomarkers associated with early-stage endometrial cancer and comparing these markers with routine tumor markers.

Although the complex physiological roles of LCN2 have been partially elucidated recently, its role in cancer, particularly, how it interacts with iron through specific mechanisms, is not fully understood. The roles of LCN2 and MMP9 in the tumor microenvironment are intricate. LCN2 has significant effects on regulating the sensitivity of certain cells to signaling pathway inhibitors. Many human tumors express substantial amounts of LCN2. Additionally, LCN2 may influence the response to some therapeutic agents in cancer treatment.

Both MMP9 and LCN2 are involved in tumor invasion and metastasis. LCN2 may influence MMP9 activity, thereby affecting the degradation of the extracellular matrix and facilitating cancer cell migration. The positive correlation between LCN2 and MMP9 levels in tumors suggests a synergistic effect on promoting invasiveness. LCN2's role in modulating MMP9 activity could involve regulatory pathways affecting matrix degradation and cellular motility. Understanding how LCN2 regulates MMP9 and vice versa can provide insights into tumor progression and potential therapeutic targets. LCN2 can influence iron metabolism by binding iron-bound molecules, which might impact ferritin levels and availability. High LCN2 levels could affect iron distribution and storage, potentially altering ferritin levels and iron availability for tumor cells. The relationship between LCN2 and ferritin in cancer is complex. Elevated ferritin levels might indicate increased iron stores, which can support tumor growth, while LCN2 might modulate this process by affecting iron uptake and utilization. Since ferritin regulates iron storage, changes in ferritin levels could impact MMP9 activity. High iron levels might enhance MMP9 activity, promoting extracellular matrix degradation and facilitating tumor invasion. Assessing ferritin and MMP9 levels together could provide insights into the tumor's iron status and its potential for invasion and metastasis. This could aid in developing strategies for monitoring disease progression and designing targeted therapies. The interplay between MMP9, LCN2, and ferritin in cancer is complex and multifaceted. Understanding their interactions can reveal mechanisms underlying tumor progression and metastasis. Targeting these molecules could offer new therapeutic approaches to manage cancer more effectively. Future research should focus on elucidating these interactions and their implications for cancer diagnosis and treatment.

## Conclusion

Serum tumor biomarkers such as CEA, Ca19-9, Ca125, and Ca15-3 can be used not only for the supportive diagnosis of endometrial cancer but also for predicting survival and prognosis. Because the serum levels of these commonly used tumor markers are significant prognostic factors and indicators of therapeutic effect and recurrence in cancer patients, they provide clinical benefit in the differential diagnosis of malignant tumors, monitoring the disease, and evaluating treatment efficacy. However, due to the highly heterogeneous nature of endometrial cancer and the lack of significant differences in marker levels in the early stages of the disease, these markers may not always represent adequate standards for accurate diagnosis. There is a need for sensitive and specific biomarkers, especially for early-stage cases. Based on our study results, LCN2, MMP9, and ferritin were concluded to be practical markers in early cases. Serum LCN2 and MMP9 levels could be good clinical tools for supportive diagnosis of early-stage endometrial cancer. Ferritin was also significantly sensitive. Therefore, the combined detection of these markers may be more beneficial for cancer diagnosis.

There is a need for sensitive and specific biomarkers, especially for early-stage cases. Our study concluded that LCN2, MMP9, and ferritin are practical markers in early cases. Serum LCN2 and MMP9 levels could be good clinical tools for the auxiliary diagnosis of early-stage endometrial cancer. Ferritin was also significantly sensitive. Therefore, the combined detection of these markers may be more beneficial for cancer diagnosis.

## Abbreviations

EC: Endometrial cancer
LCN2: Lipocalin-2
MMP9: Matrix metalloproteinase-9
TIMP1: Tissue inhibitor of matrix metalloproteinase-1
IL: Interleukin
TNF-α: Tumor necrosis factor-alpha
CA125: Carbohydrate antigen 125
CA15-3: Carbohydrate antigen 15–3
CA19-9: Carbohydrate antigen 19-9
CEA: Carcinoembryonic antigen
GC: Glucocorticoid
FIGO: International Federation of Gynecology and Obstetrics
RT-PCR: Reverse transcription polymerase chain reaction
ELISA: Enzyme-linked immunosorbent assay
ROC: Receiver operating characteristic
BSO: Bilateral salpingo-oophorectomy
LVI: Lymphovascular invasion
PLND: Pelvic lymph node dissection
TAH: Total abdominal hysterectomy
BMI: Body mass index
AFP: Alpha-fetoprotein
NPV: Negative predictive value
PPV: Positive predictive value.
